# 404. COVID-19 Normality Rate: Criteria for Optimal Time to Return to In-person Learning

**DOI:** 10.1093/ofid/ofab466.605

**Published:** 2021-12-04

**Authors:** Carlos Starling, Jackson Machado-Pinto, Unaí Tupinambás, Estevão Urbano Silva, Bráulio R G M Couto

**Affiliations:** 1 Sociedade Mineira de Infectologia - SMI, Belo Horizonte, Minas Gerais, Brazil; 2 Comitê de Enfrentamento à COVID-19, Prefeitura de Belo Horizonte - PBH, Belo Horizonte, Minas Gerais, Brazil; 3 Universidade Federal de Minas Gerais - UFMG, Belo Horizonte, Minas Gerais, Brazil; 4 Hospital Madre Teresa, Belo Horizonte, Minas Gerais, Brazil; 5 Centro Universitário de Belo Horizonte - UniBH, Belo Horzonte, Minas Gerais, Brazil

## Abstract

**Background:**

The COVID-19 pandemic created the most severe global education disruption in history. According to UNESCO, at the peak of the crisis over 1.6 billion learners in more than 190 countries were out of school. After one year, half of the world’s student population is still affected by full or partial school closures. Here we investigated whether or not it is possible to build a multivariate score for dynamic school decision-making specially in scenarios without population-scale RT-PCR tests.

**Methods:**

Normality rate is based on a COVID-19 risk matrix (Table 1). Total score (TS) is obtained by summing the risk scores for COVID-19, considering the six parameters of the pandemic in a city. The COVID-19 Normality Rate (CNR) is obtained by linear interpolation in such a way that a total score of 30 points is equivalent to a 100% possibility of normality and, in a city with only six total points would have zero percent chance of returning to normality: CNR = (TS – 6)/24 (%). The criteria for opening and closing schools can be defined based on the percentages of return to normality (Table 2).

Table 1. Limits for each parameter of the risk matrix and "normality" scores in relation to COVID-19: the lower the risk, higher is the “normality” score.

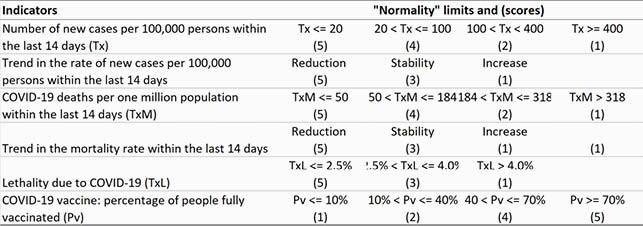

Table 2. Criteria for opening and closing schools in a city according to the COVID-19 Normality Rate.

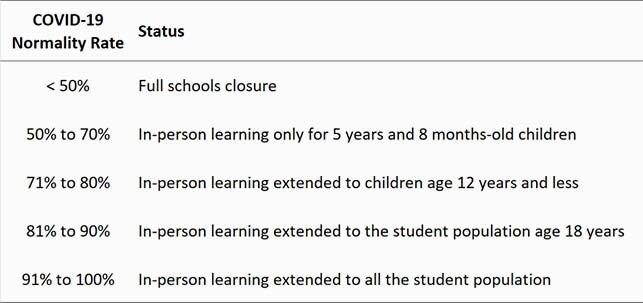

**Results:**

at June 3rd, 2021, we evaluated all 5,570 Brazilian cities (Figure 1): 2,708 cities (49%) with COVID-19 normality rate less than 50% (full schools closure), 2,223 cities (40%) with normality rate between 50% and 70% (in-person learning only for 5 years and 8 months-old children), 583 with normality rate between 71% and 80% (in-person learning extended to children age 12 years and less), 583 cities (1%) with normality rate between 81% to 90% (in-person learning extended to the student population age 18 years), and just one city with 92% COVID-19 normality rate (in-person learning extended to all the student population). We calculated the COVID-19 normality rate between January and May, 2021, in four countries: Brazil, USA, UK, and Italy (Figure 2). At Jun, 3^rd^, 2021, percentage of people fully vaccinated in Brazil varied from 0% to 69%, an average of 11%.

Figure 1. COVID-19 Normality Rate in 5,570 cities in Brazil, Jun/03/2021.

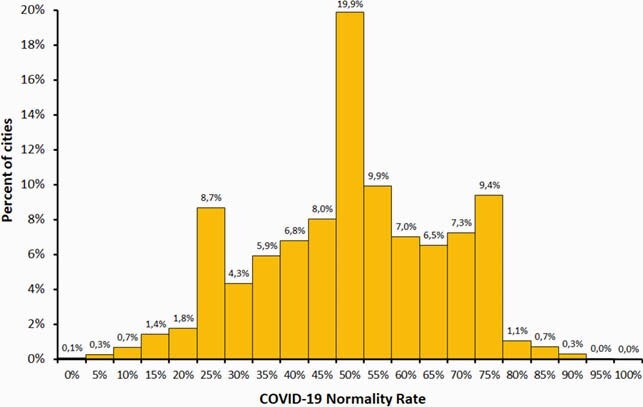

Figure 2. COVID-19 Normality Rate between January and May, 2021: comparison among Brazil, USA, UK, and Italy.

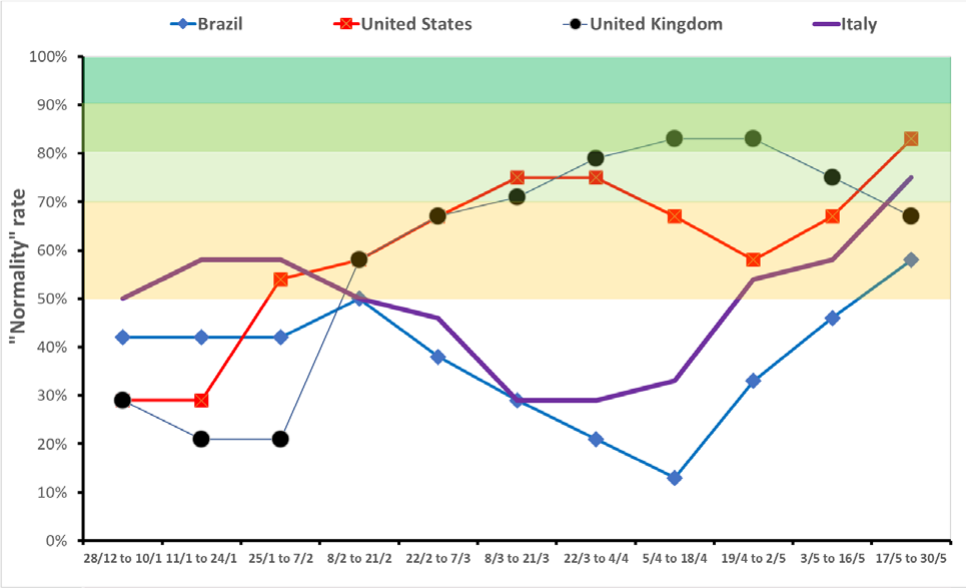

**Conclusion:**

COVID-19 vaccination programs take several months to implement. Besides fully vaccination of the population, it is important to check if people became really safe from the virus. The COVID-19 Normality Rate is a double check multivariate score that can be used as a criteria for optimal time to return to in-person learning safely.

**Disclosures:**

**All Authors**: No reported disclosures

